# Abrupt climate changes in the last two deglaciations simulated with different Northern ice sheet discharge and insolation

**DOI:** 10.1038/s41598-021-01651-2

**Published:** 2021-11-25

**Authors:** Takashi Obase, Ayako Abe-Ouchi, Fuyuki Saito

**Affiliations:** 1 Atmosphere and Ocean Research Institute, The University of Tokyo, 5-1-5, Kashiwanoha, Kashiwa, Chiba 277-8568 Japan; 2 National Institute of Polar Research, Tachikawa, Japan; 3 Japan Agency for Marine-Earth Science and Technology, Yokohama, Japan

**Keywords:** Palaeoceanography, Palaeoclimate

## Abstract

There were significant differences between the last two deglaciations, particularly in Atlantic Meridional Overturning Circulation (AMOC) and Antarctic warming in the deglaciations and the following interglacials. Here, we present transient simulations of deglaciation using a coupled atmosphere–ocean general circulation model for the last two deglaciations focusing on the impact of ice sheet discharge on climate changes associated with the AMOC in the first part, and the sensitivity studies using a Northern Hemisphere ice sheet model in the second part. We show that a set of abrupt climate changes of the last deglaciation, including Bolling–Allerod warming, the Younger Dryas, and onset of the Holocene were simulated with gradual changes of both ice sheet discharge and radiative forcing. On the other hand, penultimate deglaciation, with the abrupt climate change only at the beginning of the last interglacial was simulated when the ice sheet discharge was greater than in the last deglaciation by a factor of 1.5. The results, together with Northern Hemisphere ice sheet model experiments suggest the importance of the transient climate and AMOC responses to the different orbital forcing conditions of the last two deglaciations, through the mechanisms of mass loss of the Northern Hemisphere ice sheet and meltwater influx to the ocean.

## Introduction

The climate system of the Earth underwent glacial–interglacial cycles associated with growth and decay of continental ice sheets, driven by insolation changes as the external forcing, and by climate system feedbacks. The transition from the glacial state to the interglacial state (deglaciation) typically occurred within about 10,000 years, characterized by global warming and melting of continental ice sheets^1–3^. Based on geological reconstructions from multiple lines of evidence, there were significant differences in the climate events of the last two deglaciations and the climate states of the following interglacials^4^. In particular, during the last deglaciation (glacial Termination 1, ~ 19 to 11 ka BP, hereafter referred to as T1), there was abrupt strengthening in the Atlantic Meridional Overturning Circulation (AMOC), which caused the Bolling–Allerod (BA) warming event during the middle stage^5,6^. After the BA warming, the climate of the Antarctic region followed a cooling trend known as Antarctic Cold Reversal (ACR, 14.5 to 12.8 ka BP), followed by the Younger Dryas (YD, 12.8 to 11.6 ka BP), characterized by weakening in the AMOC and Antarctic warming until the onset of the present interglacial. In contrast, during the penultimate deglaciation (glacial Termination 2, ~ 138 to 128 ka BP, hereafter T2^4^), the AMOC was weak for about 4000 years (~ 133 to 129 ka BP) during the middle and later stage of T2, followed by the strengthening of AMOC in the last interglacial (LIG, ~ 129 to 116 ka BP)^7–10^. There were significant differences in the climate state of this interglacial compared with the present interglacial, as the sea surface temperature (SST) of the Southern Ocean and surface temperature over the Antarctic continent during the LIG were about 2° higher than those of the present interglacial^11^. There were also differences in the global mean sea level, which was higher during the LIG than in the present by at least 5 m^12–14^, presumably because of mass loss of the West Antarctic Ice Sheet (WAIS)^15,16^. It has been noted that a weakened AMOC during the early LIG caused the Antarctic warmth^17,18^ as the longer duration of weakened AMOC contributed to Antarctic warming^19–22^. Moreover, it has been hypothesized that greater boreal summer insolation caused faster retreat of Northern Hemisphere ice sheets and thus caused a weak AMOC throughout T2^8,9,23,24^ based on the reconstructed sea level rise of T2, indicating that it was significantly faster than that of T1^8^. Therefore, it is of importance to investigate the role of orbital forcing on different climate events during the last two deglaciations through melting of Northern Hemisphere ice sheets and AMOC changes.

Transient simulations of the last deglaciation have been conducted using climate models, and climate events associated with variations in the AMOC have been represented by applying meltwater flux to the ocean^25,26^. Clark et al.^27^ conducted transient simulation of T2 using reconstructed climate forcing and greater meltwater flux than T1. They compared the results with the simulation of T1^25^ and showed that the greater meltwater flux of T2 corresponding to Heinrich event 11 led to longer weakening in the AMOC and significant Antarctic warming that was sufficient to cause the mass loss of the WAIS. However, it is still unclear which phase of the deglaciation was important for the AMOC and Antarctic warming, and what the cause of significant ice sheet retreat during deglaciation was. Recently, abrupt AMOC recovery and associated ACR have been simulated with constant meltwater flux by transient simulation of the last deglaciation using the atmosphere–ocean coupled General Circulation Model (GCM) MIROC (Ref.^28^, hereafter OA2019).

In this study, first, we conducted sensitivity experiments of deglaciation with different levels of meltwater influencing the AMOC and surface temperature mainly over the Antarctic region, using an atmosphere–ocean coupled GCM. Second, we conducted sensitivity experiments of the effect of insolation on melting of the Northern Hemisphere ice sheets, using a three-dimensional Northern Hemisphere ice sheet model. We discuss the roles of orbital forcing and climate system feedbacks in the differences of the last two glacial terminations and the following interglacials.

## Results of transient climate model deglaciation experiments

**Figure 1:**
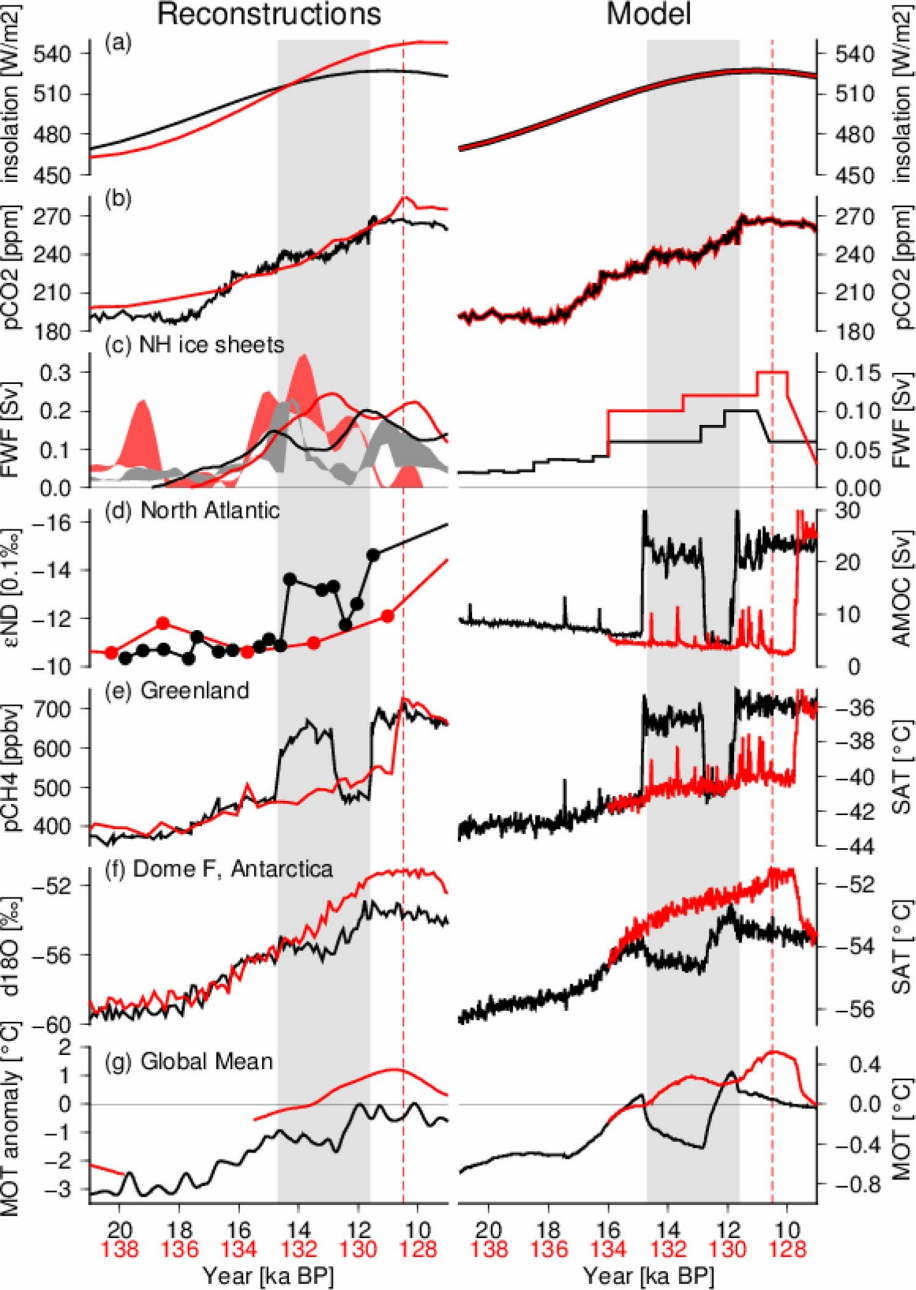
Climate model experimental design and results (right panels) compared with reconstructions (left panels). The black and red lines represent the last and penultimate deglaciations, respectively. The grey shaded areas correspond to the period of the last deglaciation from BA to onset of the Holocene (14.7 to 11.6 ka BP). The vertical red dashed lines (128.5 ka BP) correspond to the onset of the Last Interglacial, following^4^. Left panels: (**a**) Summer solstice insolation at 65° N^36^. (**b**) Atmospheric CO_2_ concentrations^37^. (**c**) Northern Hemisphere ice sheet volume loss rate from two ice sheet reconstructions (grey and red shaded bands^4,31^) and a three-dimensional dynamic ice sheet model (bold lines^38^). The volume loss rates are calculated from ice sheet volume changes during intervals of 1000 years. (**d**) Neodymium isotopes of the North Atlantic as a proxy of the relative influences of North Atlantic Deep Water and Antarctic Bottom Water, which is an indicator of the AMOC^9,35^. (**e**) Atmospheric CH_4_^39^, which reflects abrupt AMOC changes during deglaciations. (**f**) Antarctic oxygen isotopes at Dome Fuji^34^. (**g**) Global mean ocean temperature based on noble gases (splined “Mix” of Refs.^24,40^). All ice core records are on the AICC2012 age scale. Right panels: (**c**) Applied freshwater flux in the North Atlantic in two experiments. (**d**) AMOC, defined as the maximum meridional streamfunction of the AMOC between 30° N and 90° N and below 500 m depth. (**e**) Annual mean 2-m air temperature in Greenland (3-point average of GISP2, NGRIP, and NEEM ice cores). (**f**) Annual mean 2-m air temperature at Dome Fuji, Antarctica. (**g**) Global mean ocean temperature. The time series of model results were generated from 20-year averages.

In this section, we describe the results of two deglaciation experiments. In Fig. 1, the reconstructions, experimental design and results from the two deglaciation experiments are compared by offsetting the age of T2 by 118 ka to match the phases of deglacial climate changes, in the same manner as Clark et al.^27^. The simulation of T1 (T1-like experiment) is from OA2019^28^, and was extended to 9 ka BP to serve as a T1-like experiment covering the YD event and onset of the Holocene. In the sensitivity experiment to imitate T2 (T2-like experiment), we changed meltwater flux while maintaining identical orbital and atmospheric greenhouse gas forcings of T1 (Fig. 1a,b). In the T2-like experiment, about 1.5 times more meltwater was applied (Fig. 1c right side red lines), which roughly corresponded to the sea level rise rates of T1 and T2 during the middle stages of the deglaciations based on reconstructions (Fig. 1c left side shading). We used the ice sheet reconstructions^29,30^ of Ref.^31^, and the sea level and meltwater based on ice-rafted debris^32,33^ of Ref.^4^. The values were carefully estimated so that the total amounts of applied meltwater in the two experiments did not exceed the amounts estimated from the reconstructions (Supplementary Fig. S1). We conducted several sensitivity experiments and found that the timing of AMOC changes was sensitive to the meltwater flux; a greater amount of meltwater tended to weaken the AMOC by reducing the sea surface salinity of the North Atlantic (Supplementary Fig. S2). We chose one experiment to serve as the T2-like experiment, which exhibited similar time series of the reconstructed AMOC and Antarctic air temperature of T2 (Refs.^9,34^; Fig. 1d–f). The time series of the simulated AMOC changes were compared with relative enrichment in heavy neodymium isotopes (Fig. 1d), which is a proxy of the relative influence of water mass from the North Atlantic Deep Water and Antarctic Bottom Water^9,35^, in the same manner as previous studies^24,27^.

In the T1-like experiment, an abrupt increase in the AMOC occurred (around 15 ka) in response to gradual warming even under continuous meltwater (Fig. 1d). As discussed in OA2019, this abrupt increase in the AMOC with gradual background climate change has been indicated by several climate models^41–44^. The exact mechanisms of the abrupt increase are still under investigation, but based on OA2019, the gradual warming tended to change the density of seawater, which is critical to the retreat of sea ice and recovery of deep water formation in the North Atlantic. The changes of the simulated Greenland temperatures in T1 and T2 closely matched the changes of the AMOC, consistent with atmospheric methane records (Fig. 1e), which represent climate changes in the Northern Hemisphere and methane sources and sinks^39^. The AMOC returned to a weak mode in response to increased meltwater during the YD, and reached a vigorous mode at the end of the YD (about 11.6 ka BP, Fig. 1d). This reduction in AMOC in the YD can be simulated without reduction in meltwater, because of the oscillatory nature of the AMOC (Supplementary Fig. S2 pink lines). In contrast, in the T2-like experiment, the AMOC maintained a weak mode throughout the deglaciation until 128 ka BP, as the larger amount of meltwater reduced the salinity and prevented deep convection in the North Atlantic. In Antarctica, there was cooling in the T1-like experiment in response to abrupt increase in the AMOC (15–13 ka in Fig. 1d,f), which closely corresponded to ACR. The duration or cooling of ACR tended to be shorter than reconstructions in previous deglaciation experiments with other climate models^45^, but in our simulation, the Antarctic cooling continued for about 2000 years until the onset of the YD event, as in reconstructions. In contrast, surface air temperature (SAT) around Antarctica continued warming in the T2-like experiment until 128 ka BP, and the simulated peak in Antarctic SAT was higher than the interglacial level by about 2° (Fig. 1f). The YD event associated with weakening of the AMOC in the T1-like experiment turned into an Antarctic warming trend for about 1300 years, but the Antarctic SAT did not reach the level of the T2-like experiment. Interestingly, a similar trend was found in the global mean ocean temperature, with a decrease during ACR of the last deglaciation and a maximum value during the onset of the LIG that was larger than the level of the Holocene^24,40^. These trends in global mean ocean temperature in response to the AMOC can be explained by subsurface and deep ocean temperature changes, which exhibited cooling after a recovery of the AMOC (Supplementary Fig. S3), as in previous climate modelling studies^46–48^. The response of the subsurface ocean to AMOC change was relatively rapid; the increase in the AMOC produced significant cooling within a few 100 years (Supplementary Fig. S4a,b black lines). The response of the deep-ocean was more gradual (Supplementary Fig. S4c). The amplitudes of simulated temperature changes from the glacial to the interglacial were smaller than those of reconstructions (Fig. 1f,g), because of the experimental design, wherein the ice sheet was fixed to that of the Last Glacial Maximum (LGM). Nevertheless, the results clearly show that the different temporal patterns of warming and cooling trends of the Antarctic SAT and Southern Ocean SST of T1 and T2 can be simulated based only on the difference in meltwater in the North Atlantic.

**Figure 2:**
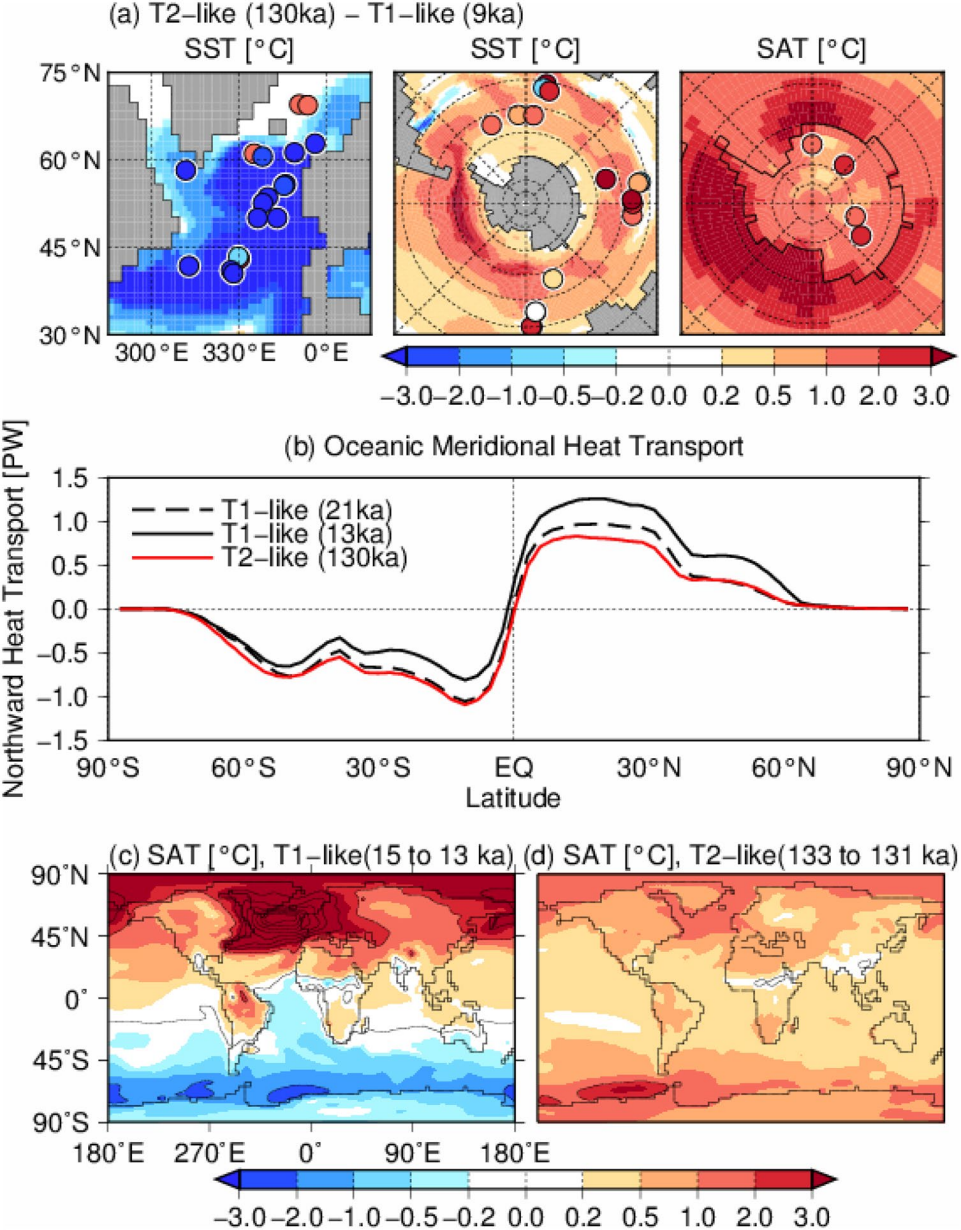
(**a**) SST and SAT difference between the T2-like experiment at 130 ka BP in relation to the T1-like experiment at 9 ka, corresponding to the Holocene level. The circles represent reconstructions (anomaly of 130 ka BP relative to the present^11^). (**b**) Simulated oceanic meridional heat transport (positive indicates northward heat transport) at 21 ka and 13 ka in the T1-like experiment and at 130 ka in the T2-like experiment. (**c**) SAT change during in the T1-like (15–13 ka BP) and T2-like (133–131 ka BP) experiments, corresponding to the Antarctic Cold Reversal of T1. The maps were generated using GMT version 4.5.9 (URL: https://www.soest.hawaii.edu/gmt/).

**Figure 3:**
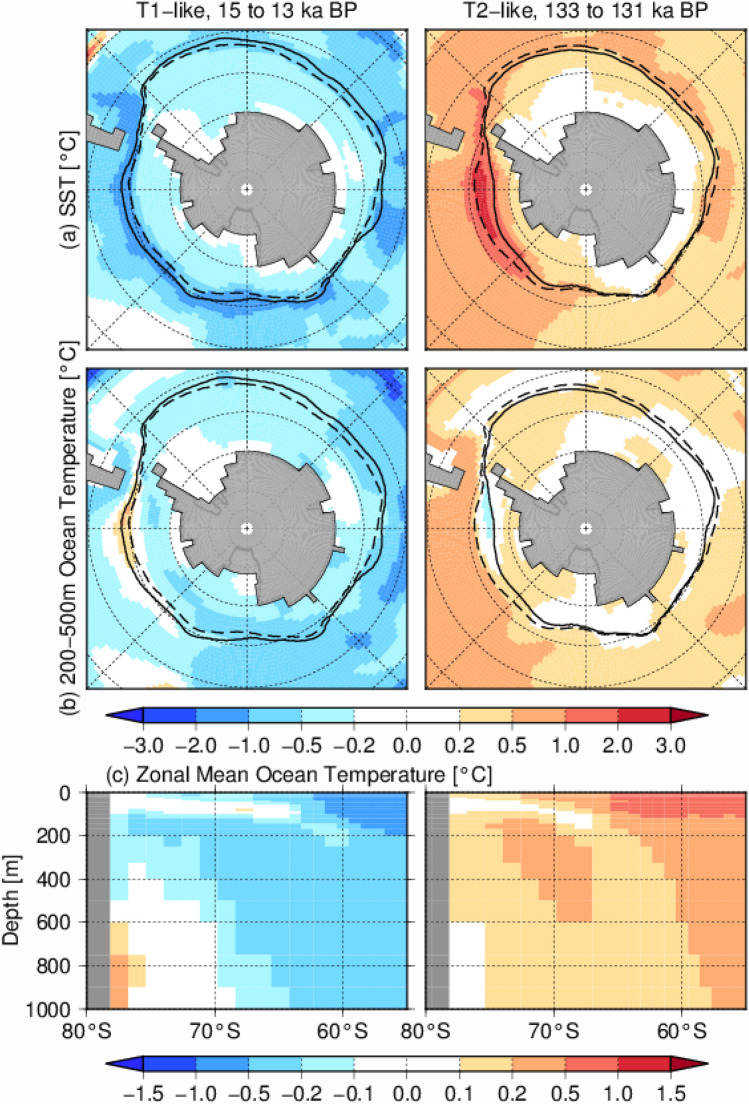
Ocean temperature changes in the T1-like (15–13 ka BP) and T2-like (133–131 ka BP) experiments, corresponding to the Antarctic Cold Reversal of T1. (**a**,**b**) SST and subsurface ocean temperature averaged at 200–500 m depth. Dashed and bold lines indicate austral winter sea ice extent, defined by a sea ice concentration of 15%, at 15 and 13 ka (left panels), and 133 and 131 ka (right panels), respectively. (**c**) Zonal mean ocean temperature averaged for the whole of Antarctica. The maps were generated using GMT version 4.5.9 (URL: https://www.soest.hawaii.edu/gmt/).

In Fig. 2a, Antarctic SAT and SST at 130 ka from the T2-like experiment are compared with the reconstruction of 130 ka BP, which corresponds to the early LIG^11^. The model results in Fig. 2a indicate the temperature anomaly at the early stage of the last interglacial (130 ka), relative to the early Holocene (9 ka). The SAT and SST of the T2-like experiment are characterized by a cooler climate over the Northern Hemisphere and a warmer climate in the Southern Hemisphere (Fig. 2a), and the magnitude of warming in the Southern Ocean and Antarctica is comparable to the reconstructed temperature anomalies during the early LIG. The increase in the AMOC that occurred in the T1-like experiment increased northward heat transport, whereas the heat transport of the T2-like experiment at 130 ka BP was almost the same as or less than that in the LGM (Fig. 2b). These results indicate that the heat accumulated in both hemispheres in the T2-like experiment because of the sustained weak AMOC, whereas the abrupt increase in the AMOC of the T1-like experiment induced bipolar climate changes (Fig. 2c). The SST and subsurface ocean temperature, which are important for the mass balance of ice shelves^27,49–52^, showed a cooling trend and the sea ice extent increased during 15 to 13 ka BP in the T1-like experiment (Fig. 3). In contrast, there was warming and sea ice retreat in the T2-like experiment; therefore, the warmer Antarctic climate in the T2-like experiment likely contributed to negative mass balance of the Antarctic ice sheet. At the same time, the retreat of sea ice in the T2-like experiment promoted vertical convection in the Southern Ocean and resulted in a greater increase in SST and cooling in the subsurface of the Amundsen Sea, a similar pattern to that reported by Pedro et al.^53^ but in a different ocean basin (Supplementary Fig. S5).

In Supplementary Fig. S6, we present an additional deglaciation experiment using realistic orbital and greenhouse gas forcing of T2. In this experiment, the three orbital parameters (obliquity, eccentricity and perihelion) and greenhouse gas conditions were changed to those of T2 at 138 ka from those in the T1 at 20 ka. The meltwater flux was set to the same level of T1 in the early stage of the deglaciation, and it was increased to 0.08 and 0.1 Sv (10^6^ m^3^/s) at 133 and 132 ka respectively; the meltwater flux was at the same level as in the T2-like experiment (Supplementary Fig. S6e). The results show that the abrupt increase in the AMOC occurred before 133 ka, but the AMOC returned to a weak mode in response to meltwater, and the weak AMOC remained until the onset of the last interglacial (Supplementary Fig. S6f). In particular, the AMOC oscillated twice during the early stage of T2 (135–133 ka), possibly corresponding to the abrupt climate changes recorded by dust concentrations in an Antarctic ice core from Dome Fuji^34^ or the abrupt changes in the methane record and SSTs in the North Atlantic before 133 ka^4,54,55^. The stronger AMOC in the early stage of the deglaciation was likely because of the orbital parameters and atmospheric CO_2_ forcing, which tend to warm temperatures over the North Atlantic and the Antarctic region. The most important result in this study was that the Antarctic temperature of T2 was still higher than that of the T1-like experiment (Supplementary Fig. S6g) even if there was an Antarctic cooling event in the early stage of the deglaciation. These results indicate that a weak AMOC during the middle and later stages of the deglaciation was sufficient for the Antarctic warmth of T2. This can be explained by the fact that the typical response time of Antarctic warming to the AMOC is about 1000 to 2000 years^22,48,56,57^.

## Results of ice sheet model experiments

**Figure 4:**
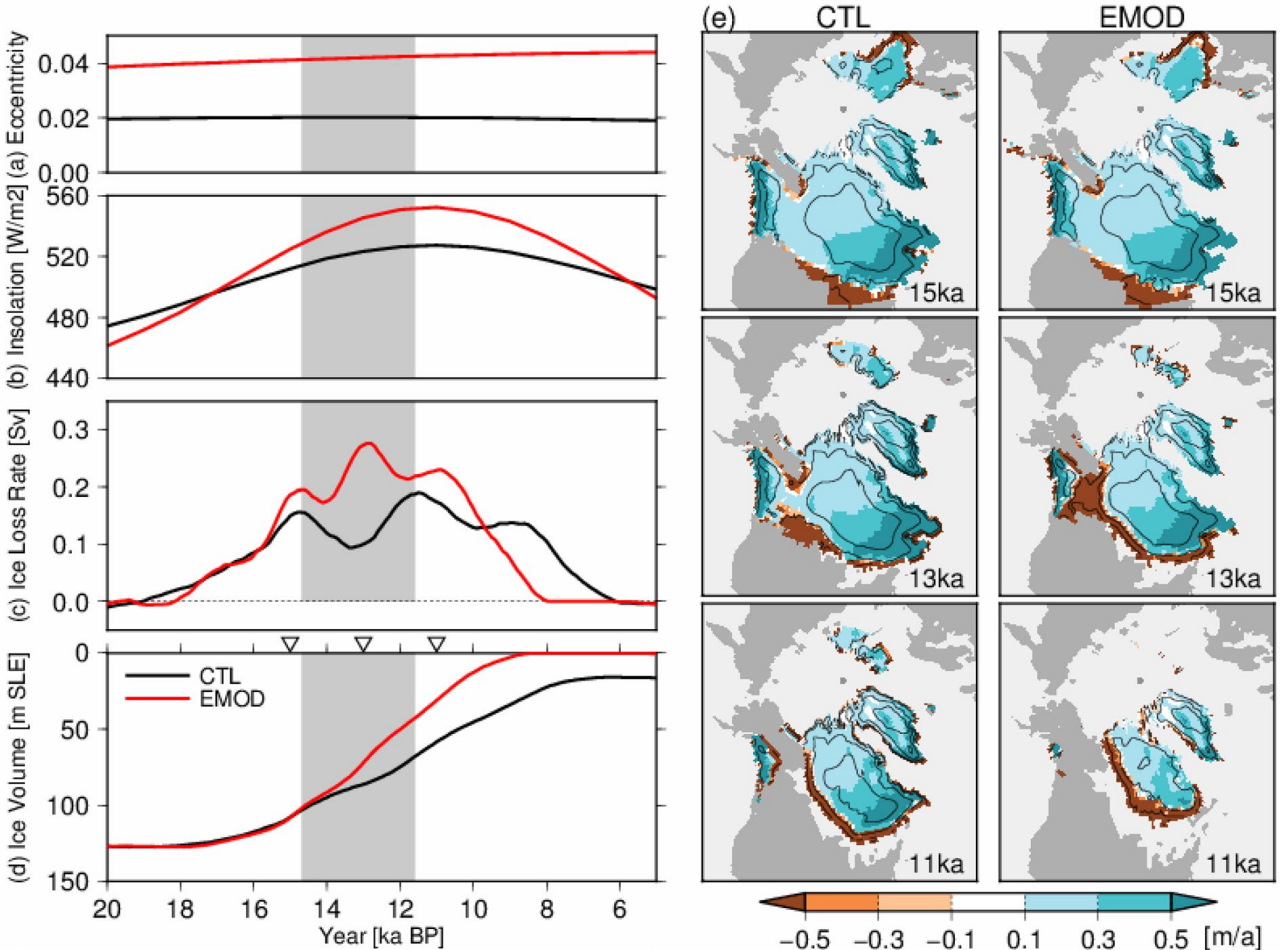
Experimental design and results of the ice sheet model experiments. The black lines indicate the results of simulation of the last 400,000 years^38^. The grey shaded area corresponds to the last deglaciation from the BA period to onset of the Holocene (14.7 to 11.6 ka BP), as in Fig. 1. The red lines indicate the experimental design and results of the EMOD (Eccentricity MODified) experiment, where orbital eccentricity was changed to that of T2 at the time of 20 ka BP. (**a**) Orbital eccentricity and (**b**) summer solstice insolation at 65° N using each eccentricity of (**a**). (**c**) Simulated Northern Hemisphere ice loss rate, calculated based on the ice volumes changes in 1000-year intervals. (**d**) Volume of Northern Hemisphere ice sheets, calculated based on the sum of the North American and Eurasian ice sheets. Here, the volumes of the ice sheets (m^3^) were divided by 4.0 × 10^14^ to determine the sea level equivalent^58^. Triangles show the time slices of (**e**). (**e**) Map showing shapes and surface mass balances of ice sheets at three different time slices. Colours indicate the surface mass balance in metres per year. The maps were generated using GMT version 4.5.9 (URL: https://www.soest.hawaii.edu/gmt/).

To investigate the possible cause and mechanism of the difference in the meltwater from Northern Hemisphere ice sheets in the later stage between the two glacial terminations, we conducted sensitivity studies using a Northern Hemisphere ice sheet model IcIES-MIROC^38^. Note that the following experiments with this ice sheet model were independent of the climate model deglaciation experiments in this study. In the sensitivity experiment (red line in Fig. 4a, EMOD experiment), the orbital eccentricity was changed to that of the penultimate deglaciation (same period as T2) from the last deglaciation (same period as T1) using the 20 ka ice sheet configurations of the model as an initial condition. In this setting, the larger eccentricity of T2 relative to T1 contributed to greater summer insolation in the Northern Hemisphere and negative mass balance of the ice sheets only after the middle stage of deglaciation (about 17 ka BP), as the climatic precession was negative in the early deglaciation (Fig. 4b). The simulated Northern Hemisphere ice sheet volume loss rate was very close to that of the control experiments in the early deglaciation until about 16 ka BP (Fig. 4c,d). However, significant differences occurred after the middle stage of the deglaciation; the greater orbital eccentricity of the EMOD experiment compared with the control experiment induced greater summer insolation and negative mass balance of the ice sheets after 16 ka BP (Fig. 4e). The rate of ice sheet volume change was ~ 1.5 times larger than that of the control experiment during the period of 15 to 12 ka BP, which corresponds to the BA period of T1. Therefore, the greater orbital eccentricity of T2 compared with T1 had significant influence on the mass balance of Northern Hemisphere ice sheets during the middle and later stages of deglaciation, and also on the meltwater flux caused by volume loss of ice sheets.

## Discussion

The climate model results can be summarized as follows: the middle to late stages of the two deglaciations (133 to 129 ka and 15 to 11 ka, grey shaded area in Fig. 1) show a substantial difference in the occurrence of abrupt changes of AMOC and bipolar seesaw climate change because of the meltwater discharge from the Northern Hemisphere ice sheets (by factor of 1.5). Together with previous studies, these results can be explained by the following mechanism. First, the gradual warming of the last two deglaciation tended to strengthen the AMOC as reported by OA2019^28^, but a relatively small amount of meltwater in the last deglaciation, supported by sea level reconstruction^8^, enabled the AMOC to enter a vigorous mode earlier than the interglacial onset in T1, as in BA warming. Second, the level of meltwater during the latter half of the deglaciation was crucial for Antarctic temperature in T2 and the LIG; it could be higher than in T1 because of a weakened AMOC during the later stage of the deglaciation of T2 (Supplementary Fig. S6g). These results are consistent with a previous study wherein a longer duration of meltwater formation in the deglaciation was found to be needed for Antarctic warming of the LIG^22^. Early AMOC recoveries might not occur as in the reconstructions if meltwater corresponding to Heinrich event 11 is applied^8^. Third, Antarctic warming was interrupted by BA warming and ACR during the later stage of T1, which was critical for a lower Antarctic temperature compared with T2. In our results, Antarctic temperature exhibited a cooling trend for about 2000 years during ACR, as in reconstructions, and the Antarctic warming during the YD event was not sufficient to catch up with the warming exhibited in the T2-like experiment (Fig. 1f). The sustained weak AMOC until the end of deglaciation led to significant Antarctic warming during the onset of the LIG compared with the Holocene, which is consistent with previous modelling studies^27,43^. Thus, our results highlight the importance of transient responses of the climate system dynamics of deglaciation to gradual changes of ice sheet discharge and insolation, associated with AMOC affecting Antarctic warming.

Clark et al.^27^ suggested that the larger Eurasian ice sheet during the Penultimate Glacial Maximum (PGM) before T2^59,60^ was one primary source of meltwater during the early stage of T2. The dynamics of marine ice sheets and the extensive Eurasian ice sheet at the PGM were not represented in our ice sheet model, but it is important that the land-based ice sheet alone could be a source of differences after the middle stage of the deglaciation, when there were significant differences in the surface mass balances of the ice sheet caused by differences in insolation (Fig. 4). Moreover, the ice sheet model results indicate that the relative importance of the types of forcing could differ between the stages of the deglaciations; the role of orbital eccentricity in mass loss of Northern Hemisphere ice sheets was relatively small in the early stage, whereas it was significant in the middle stage of the deglaciations. In addition, based on the climate model results, a sustained weak AMOC during the middle and later stages was more critical to the peak Antarctic temperature during deglaciation. Even if the AMOC experienced strong modes in the early stage of the deglaciation, possibly corresponding to the observed climate fluctuations^54,55^, Antarctic temperature reached a similar thermal maximum in the late stage of the deglaciation (Supplementary Fig. S6g). Therefore, we propose that in the early stage of T2, the extensive Eurasian ice sheet was an important source of meltwater related to a stronger and longer Heinrich 11 stadial^27^, and that insolation may have been more important in the middle and later stages as a source of meltwater to sustain a weak AMOC during T2, as in reconstructions. In contrast, the climatic consequence of the last deglaciation can be interpreted to be that the smaller eccentricity of T1 could not supply significant meltwater in the North Atlantic and permitted the AMOC to recover as in reconstructions, and that this cooled the Antarctic region, with the WAIS remaining in the present interglacial. Based on the above discussion, we propose that orbital forcing was one important factor in the differences in abrupt climate events of the last two deglaciations, and in the Antarctic warmth of the following interglacials, as discussed in previous studies^8,9,23,24^.

Comparison of the last two deglaciations with climate model experiments provides a unique opportunity to investigate transient responses of the climate system to external forcing, but several climate processes in climate model simulations should be improved in the future. First, the Antarctic ice sheet configuration was fixed to that of the LGM throughout the deglaciation experiments of the climate model, which may have led to underestimation of Antarctic surface temperature changes by excluding the effect of changes in the surface elevation^19,21^. The lack of surface elevation changes affected the model–data comparison of surface temperature in the LIG, but it is of importance that the magnitude of Antarctic warming can be simulated with transient climate responses. The Northern Hemisphere ice sheets were also fixed to LGM levels; disintegration of Northern ice sheets would influence the AMOC by changing the air temperature and winds over the North Atlantic^61^. Second, the meltwater in the North Atlantic was not the same as that of the sea level reconstructions or ice sheet model results, and was simplified by applying a specific area over the North Atlantic. We used this simplification to highlight the comparison of the last two deglaciations with different levels of background meltwater, without rapid mass loss of marine ice sheets. This point can be improved further, as complex time series and distributions of meltwater by river runoff have been utilized in the simulation of the last deglaciation^25,62,63^. Third, meltwater influx into the Antarctic Ocean caused by the retreat of the Antarctic ice sheet was not considered; this meltwater could strengthen the stratification of the Southern Ocean and raise the subsurface ocean temperature at intermediate depths^26,64,65^. As shown in the last deglaciation inferred from ice-rafted debris records in the Antarctic Ocean^66^ and ice sheet modelling^67,68^, meltwater would cause greater subsurface ocean temperatures and extensive retreat of the Antarctic ice sheet during T2. In forthcoming future works, transient ice sheets and meltwater from both hemispheres will be included in the deglaciation climate model experiments. Our offline experiments with respective climate model and ice sheet model experiments highlighted the nature of climate system dynamics during deglaciation, but in the future, we plan to test transient simulations with coupled ice sheet–climate models. The LIG has been considered an important constraint of the tipping point of the WAIS^69^; therefore, understanding climate system feedback during deglaciation and interglacial stages is important as it relates to future changes. In particular, global warming and meltwater from the Greenland Ice Sheet weaken the AMOC^70^. In addition, Antarctic warming induces melting of the Antarctic ice shelves, and meltwater can induce further mass loss of the Antarctic ice sheet^71,72^. We expect that further modelling studies on transient climate–ice sheet interactions will improve our understanding of the climate system and the consequences of climate events in the past, and constrain tipping points and future loss of the Antarctic ice sheet.

## Methods

### Climate model

We used the MIROC4m AOGCM, the same model used in the simulation of the last deglaciation (Obase and Abe-Ouchi 2019, hereafter OA2019^28^). The resolution of the atmospheric component was T42 (about 2.8° × 2.8°) with 20 vertical levels, and that of the ocean component was about 1.4° × 1° with 43 vertical levels. MIROC 4 m produced vigorous AMOC under the LGM when submitted to PMIP2^73^. The coefficient of the horizontal isopycnal layer thickness diffusivity of the ocean model was changed to 7.0 × 10^6^ cm^2^/s from 3.0 × 10^6^ cm^2^/s, and the present model produces a weak AMOC under the LGM because of enhanced dense Antarctic Bottom Water (AABW) formation^34^. MIROC has been used to investigate the climate of the LGM with radiative forcing and climate feedback^74^, the effect of ice sheets on the climate and the AMOC^34,61,75^, ocean biogeochemical cycles^76–78^, and mass balance of the Antarctic ice shelves^79,80^.

The transient simulations were mostly based on the protocols proposed by the Paleoclimate Modelling Intercomparison Project Phase 4 (PMIP4) for the last and penultimate deglaciations^4,31^. Most of the experimental design of the deglaciation experiments followed OA2019, where the model was initialized with the Last Glacial Maximum (LGM) lasting for more than 30 thousand years^34^, and time-evolving insolation, atmospheric greenhouse gas concentrations, and meltwater were applied based on reconstructions^36,37,39,81^. The continental ice sheets and land–sea mask were fixed to those of the LGM (ICE-5G^82^) as used in PMIP2^83^; therefore, the climate system feedback associated with changes of ice sheets was excluded. Ice sheet reconstructions^29,30^, referred from PMIP4^31^, were used as constraints on meltwater flux in the deglaciation experiments. The meltwater was uniformly applied to 50–70° N in the North Atlantic as in previous studies^27,84^. OA2019 showed an increase in the AMOC during BA under a constant meltwater flux of ~ 0.06 Sv, and the last deglaciation experiment was extended to 9 ka BP to serve as a T1-like experiment covering the YD event and onset of the Holocene. The time series of meltwater flux in the T1-like and T2-like experiments are summarized in Supplementary Table 1. The cumulative glacial meltwater fluxes in the T1-like and T2-like experiments were 28 and 39 m sea level equivalent at 13 ka BP, and 50 and 74 m at 10 ka BP, respectively (Supplementary Fig. S1).

### Ice sheet model

The ice sheet model with the climate parameterization (IcIES-MIROC) used in this study corresponds to that described by Abe-Ouchi et al.^38^. The horizontal resolution is 1 × 1 degrees, and ice sheet dynamics are represented by the Shallow Ice Approximation. The climate factors that control the surface temperature and surface mass balance of ice sheets, such as the lapse rate, albedo feedback and stationary wave feedback, were obtained from a suite of experiments using GCM snapshots to obtain a climate parameterization^38,85^. The surface temperature distribution was parameterized as the sum of the present-day climatology and anomaly due to changes in insolation, atmospheric CO_2_, ice sheet area, and nonlinear effects estimated from climate model experiments^38^. The surface melt of the ice sheets was calculated with a positive-degree-day method^86^. The Control (CTL) experiment used an experimental design identical to that of Abe-Ouchi et al.^38^; the ice sheet model was initialized with the present-day ice sheet, and was forced by the orbital parameters^37^ and atmospheric CO_2_ concentration^87^ for the last 400,000 years (Fig. 4a). The simulated total ice sheet volume in the Northern Hemisphere largely reproduced reconstructed sea level change (Fig. 4d, black lines). A sensitivity experiment EMOD (Eccentricity MODified experiment) was performed based on the CTL experiment from 20 to 5 ka BP. In this experiment, the orbital eccentricity was changed to that of the penultimate deglaciation (133 ka BP) at the time of 20 ka BP, using the 20 ka ice sheet configurations of Abe-Ouchi et al.^38^ as an initial condition. In this setting, the larger eccentricity of T2 relative to T1 contributed to greater summer insolation in the Northern Hemisphere and negative mass balance of the ice sheets only after the middle stage of deglaciation (about 17 ka BP), as the climatic precession was negative in the early deglaciation (Fig. 4b).

## Supplementary Information

Supplementary Information.

## Data Availability

The presented model data will be available from the repository of the Center for Earth Surface System Dynamics, Atmosphere and Ocean Research Institute, The University of Tokyo (http://cesd.aori.u-tokyo.ac.jp/index_en.html). Additional data related to this paper may be requested from the authors.
