# Supplementary material for: Abrupt climate changes in the last two deglaciations simulated with different Northern ice sheet discharge and insolation
Source: Sci Rep. 2021 Nov 25;11:22359. doi: 10.1038/s41598-021-01651-2 (PMC8616927; doi:10.1038/s41598-021-01651-2)
Supplement: Supplementary file 1 — Supplementary Information. [file 41598_2021_1651_MOESM1_ESM.docx]

**Supplemental Figures:**


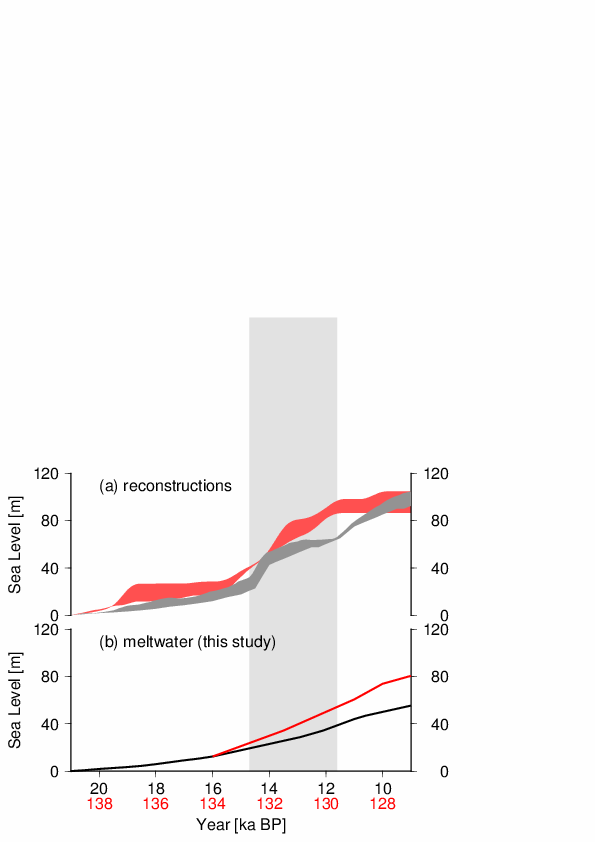


Fig. S1: Comparison of cumulative meltwater input in this study with ice sheet reconstructions, using the same datasets used in Figure 1c, shown in the form of sea level changes since the glacials (21 or 139 ka BP). (a) Grey shading indicates the last deglaciation: ice sheet volume changes from Peltier et al. (2015) and Tarasov et al. (2012), used in Ivanovic et al. (2016); red shading indicates penultimate deglaciation: sea level records (Grant et al. 2014) and ice-rafted debris datasets (Risebrobakken et al., 2007), used in Menviel et al. (2019). (b) Cumulative meltwater inputs from the T1-like and T2-like experiments in this study. The vertical grey shaded area indicates the period from Bolling-Allerod to onset of the Holocene (same as Fig. 1).


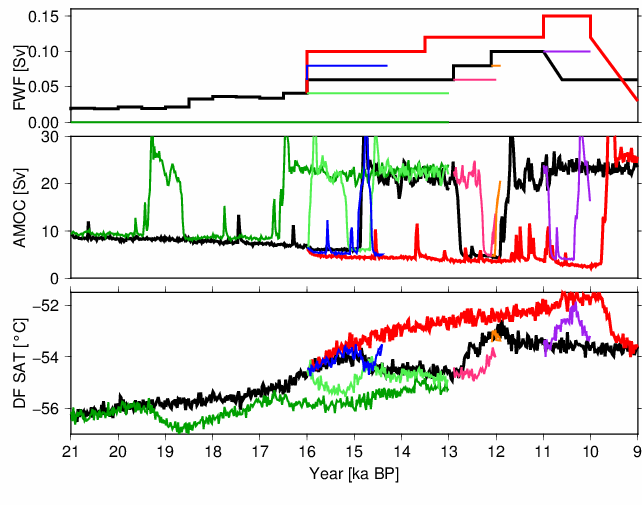


Fig. S2: Results of sensitivity experiments on meltwater flux. (a) Freshwater flux in the North Atlantic, (b) AMOC streamfunction, (c) Antarctic temperature at Dome Fuji. Lines of different colours indicate experimental design and results, and black and red lines correspond to the T1-like and T2-like experiments in Fig. 1, respectively.


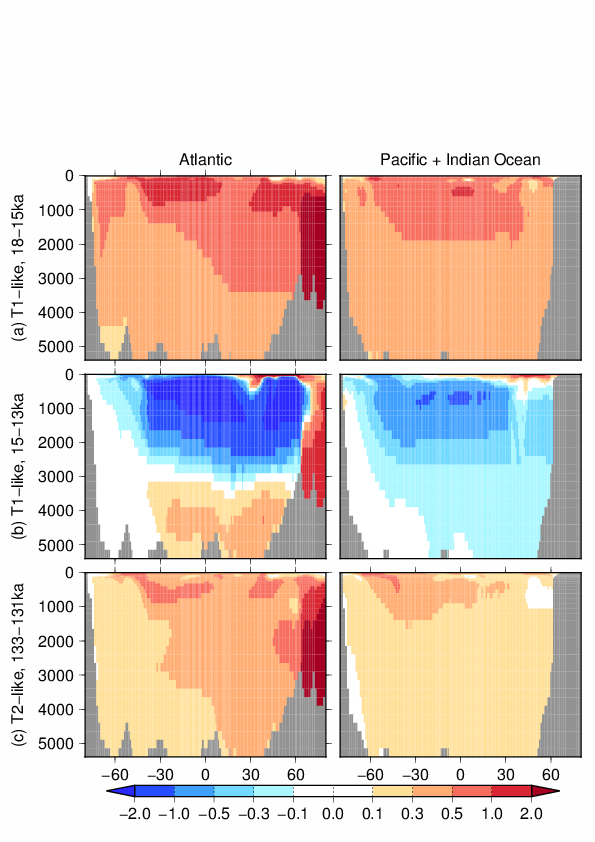


Fig. S3: Trends in zonal mean ocean temperature in each ocean basin based on the T1-like and T2-like experiments. The maps were generated using GMT version 4.5.9 (URL: https://www.soest.hawaii.edu/gmt/).


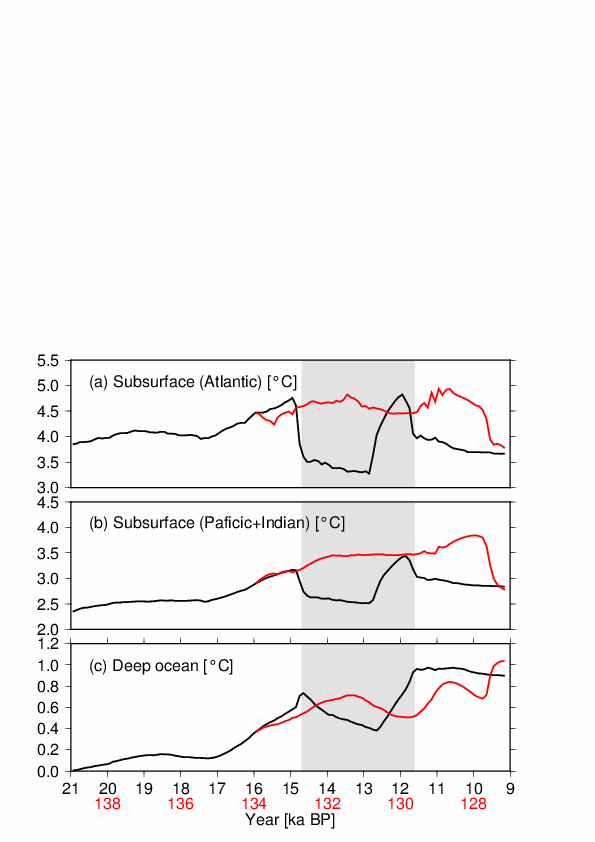


Fig. S4 Time series of mean ocean temperature in the subsurface and the deep ocean from the T1-like (black) and T2-like (red) experiments. The subsurface ocean is defined as 60°S–60°N and 500–2500 metres depth, and the deep ocean is defined as global and deeper than 2500 metres.


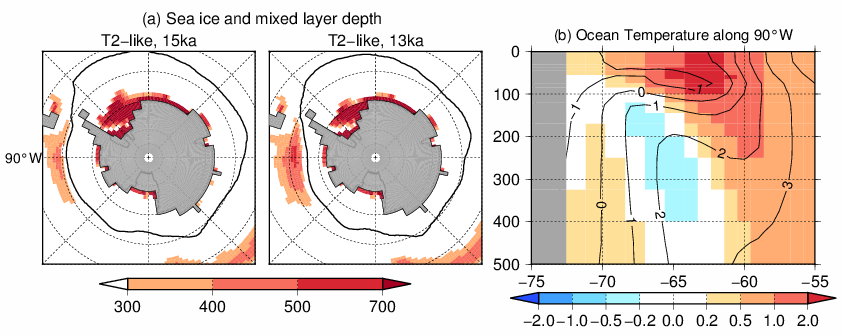


Fig. S5 (a) Winter sea ice extent (bold lines) and mixed layer depth (colours) in T2-like experiments, indicating that there was vertical convection of ~500 meters depth in the Amundsen and Bellingshausen Sea sectors of the Southern Ocean. (b) Latitude–depth diagram of ocean temperature along 90°W (Amundsen Sea) at 15 ka (contours), and trends from 15 to 13 ka BP (colours). There was strong stratification at around 65°S at 15 ka, and this stratification was weakened at 13 ka because of active vertical convection, which mixed the cold seawater at the surface and warm water at intermediate depth. The maps were generated using GMT version 4.5.9 (URL: https://www.soest.hawaii.edu/gmt/).


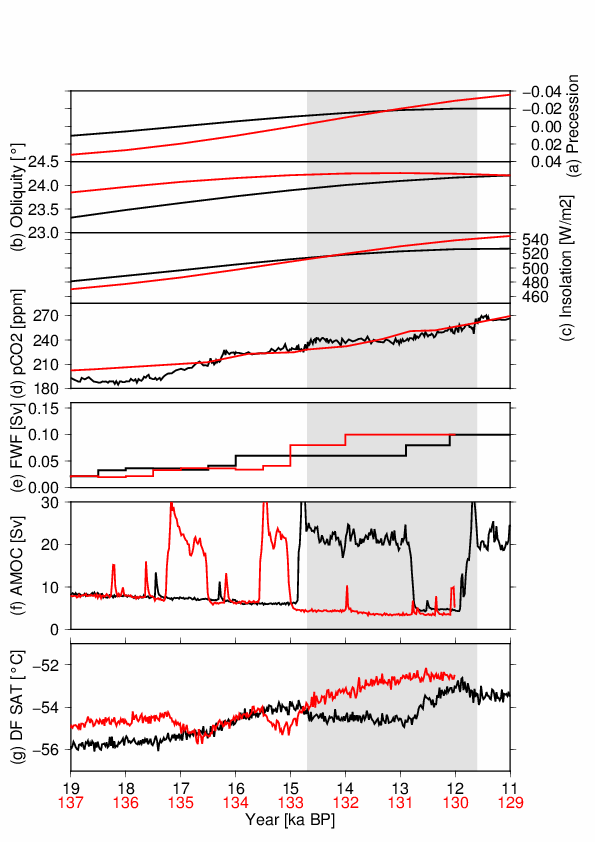


Fig. S6: Experimental design and results of additional experiments with the actual orbital and greenhouse gases of T2 (red lines). The grey shaded area indicates the period from Bolling-Allerod to onset of Holocene, and the black lines indicate the T1-like experiments same as Fig. 1. The additional experiment was initialized with the 21 ka BP, and the simulation was performed from 138 to 130 ka BP, and the results after 137 ka BP are shown in this figure. Experimental design: (a) climatic precession (b) obliquity, (c) Summer solstice insolation at 65°N, (d) atmospheric CO2 concentrations, (e) freshwater flux in the North Atlantic. (f) AMOC streamfunction, (g) surface air temperature at Dome Fuji, Antarctica.

**Supplemental Table**

| T1-like experiment | T2-like experiment |
| --- | --- |
| 21-16 ka : ice volume loss rate from Ice6G (Peltier et al. 2015) | 21-16ka (139-134ka): Same as T1 |
| 16-12.9ka: 0.06Sv | 16-13.5ka (134-131.5ka): 0.1Sv |
| 12.9-12.1ka: 0.08Sv | 13.5-11.0ka (131.5-129ka): 0.12Sv |
| 12.1-11.0ka: 0.10Sv | 11.0-10.0ka (129-128ka): 0.15Sv |
| 11.0-10.6ka: linearly decreased to 0.06Sv | 10.0-9.0ka (128-127ka):  linearly decrease from 0.12 to 0.03Sv |
| 10.6-9.0ka: 0.06Sv |  |

Table S1: Detailed time series of meltwater flux used in the T1-like and T2-like experiments
